# Molecular mechanism of Rubisco activase: Dynamic assembly and Rubisco remodeling

**DOI:** 10.3389/fmolb.2023.1125922

**Published:** 2023-02-10

**Authors:** Kazi Waheeda, Heidi Kitchel, Quan Wang, Po-Lin Chiu

**Affiliations:** ^1^ School of Molecular Sciences, Arizona State University, Tempe, AZ, United States; ^2^ Biodesign Center for Applied Structural Discovery, Arizona State University, Tempe, AZ, United States; ^3^ Laboratory of Chemical Physics, National Institute of Diabetes and Digestive and Kidney Diseases, National Institutes of Health, Bethesda, MD, United States

**Keywords:** Rubisco, Rubisco activase, carbon fixation, photosynthesis, AAA+ ATPase, redox

## Abstract

Ribulose-1,5-bisphosphate (RuBP) carboxylase-oxygenase (Rubisco) enzyme is the limiting step of photosynthetic carbon fixation, and its activation is regulated by its co-evolved chaperone, Rubisco activase (Rca). Rca removes the intrinsic sugar phosphate inhibitors occupying the Rubisco active site, allowing RuBP to split into two 3-phosphoglycerate (3PGA) molecules. This review summarizes the evolution, structure, and function of Rca and describes the recent findings regarding the mechanistic model of Rubisco activation by Rca. New knowledge in these areas can significantly enhance crop engineering techniques used to improve crop productivity.

## Introduction

Carbon assimilation is a crucial process in the global carbon cycle as well as plant photosynthesis, in which plants utilize the Calvin-Benson-Bassham (CBB) cycle to fix atmospheric carbon dioxide (CO_2_) (Bassham et al., 1950). The CBB cycle includes a series of redox reactions that convert CO_2_ into sugar compounds to maintain cell survival (Bassham et al., 1950). Ribulose-1,5-bisphosphate carboxylase-oxygenase (Rubisco) is the key enzyme in the CBB cycle (Jensen, 2000; Sharkey, 2023) and the most abundant enzyme on Earth (Ellis, 1979). It catalyzes the carboxylation of a five-carbon sugar, ribulose-1,5-bisphosphate (RuBP), and splits it into two 3-phosphoglyceric acid (3PGA) molecules (Spreitzer and Salvucci, 2002; Andersson and Backlund, 2008). Carbon fixation through Rubisco is believed to have evolved approximately 3.5 billion years ago (Nisbet et al., 2007). Now, Rubisco accounts for 95% of the fixed carbon in the biosphere (Weigmann, 2019). However, Rubisco is very inefficient with only two to ten CO_2_ molecules fixed per second (Bracher et al., 2017). This is mostly due to the Rubisco active sites being decarbamylated or occupied with intrinsic sugar phosphate inhibitors, prohibiting substrate binding for catalysis (Badger and Lorimer, 1981; Jordan and Chollet, 1983; Brooks and Portis, 1988; Orr et al., 2023).

A catalytic chaperon has co-evolved with Rubisco, the so-called Rubisco activase (Rca), which enables Rubisco function (Salvucci et al., 1985; Mueller-Cajar and Whitney, 2008; Mueller-Cajar et al., 2011; Tsai et al., 2015; Loganathan et al., 2016). Rca is an ATP-dependent enzyme that activates Rubisco by changing its conformation and promoting the dissociation of sugar phosphate inhibitors from its active sites (Portis, 2003; Andersson, 2008; Stotz et al., 2011). This mechanism increases carbamylation of Rubisco without increasing its affinity for CO_2_, meaning Rca can maximize Rubisco’s catalytic activity even when CO_2_ concentration is low (Portis et al., 1995). It has been found in rice that overexpression of Rubisco does not improve crop yield, but overexpression of Rca does (Makino and Sage, 2007; Wu et al., 2007; Suzuki et al., 2009; Fukayama et al., 2012). These results indicate that Rca is critical to Rubisco activation. A recent study showed that the overexpression of Rca interferes the electron transport within the photosystem I (Suganami et al., 2022). The function of various types of Rca in cyanobacteria and some plants has been reported, but because of its structural flexibility and polydispersity, it is challenging to generate recombinant plant Rca in high quantities for structural study. Thus, our understanding of the molecular underpinnings of Rubisco activation by Rca is limited.

This review summarizes the evolutionary, structural, and functional aspects of Rca enzyme as well as new findings about Rca and its interaction with Rubisco in recent years. Understanding the structure and function of Rca opens possibilities of enhancing crop engineering and increasing carbon fixation efficiency by improving Rubisco activation.

### Evolution of Rca

#### Rca originated in cyanobacteria

**FIGURE 1:**
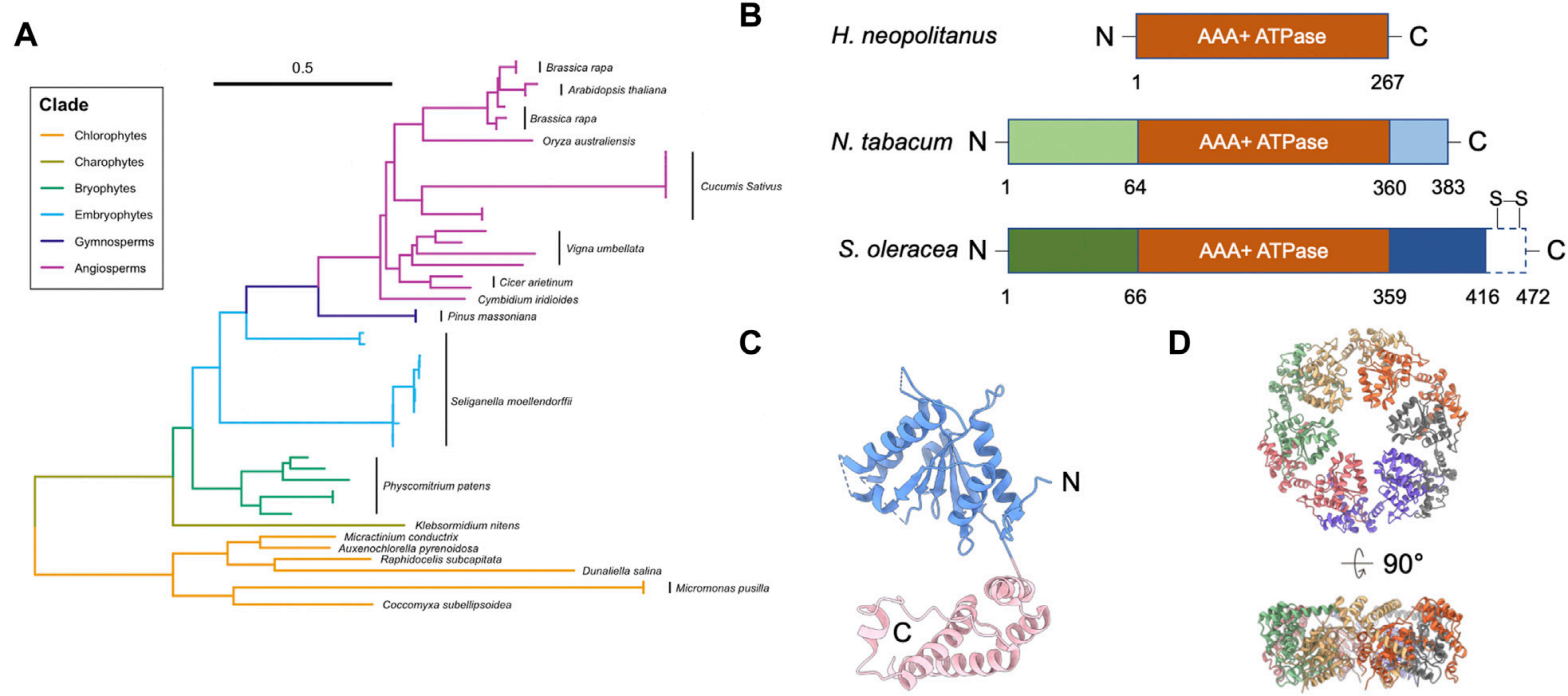
Structure of Rubisco activase (Rca). **(A)** Phylogenetic tree analysis of Rca. Scale bar indicates the branch length. Multiple Rca sequences of the same species are denoted with a black bar next to the species name. Representative Rca sequences across photosynthetic organisms, including plants and green algae, were collected using NCBI Protein BLAST; a full list of all sequence information analyzed is listed in Table 1. Sequences were aligned using ClustalW (Thompson et al., 2002), and the phylogenetic tree was generated in UGene using the Neighbor-Joining method (Saitou and Nei, 1987) and formatted using a combination of iTOL (Letunic and Bork, 2021) and RStudio software. **(B)** Domain structures of Rca from *H. neopolitanus*, *N. tabacum*, and *S. oleracea*. Dash box of the Rca of *S. oleracea* represents the C-terminal extension (CTE). Two specific redox cysteines are labelled. **(C)** Structure of tobacco Rca (PDB code: 3ZW6) (Stotz et al., 2011). Blue and pink are α/β subdomain and α-helical subdomain, respectively. Dash lines are unresolved segments. **(D)** Hexameric arrangement of the tobacco Rca. Individual monomers are in different colors.

Genetic analysis showed that the *Rca* gene likely began its evolution in cyanobacteria and remains present in all plant species (Figure 1A) (Amborella Genome Project, 2013; Nagarajan and Gill, 2018). Higher plant Rca appears to have evolved in phases, starting as a simple architecture that grew in complexity as photosynthetic mechanisms and evolutionary demands changed (Gütle et al., 2017). Recent genetic sequence analysis identified an association between environmental stress and *Rca* gene expression (Aliakbari et al., 2021). More specifically, the transition of plants from water to land is thought to have driven significant changes in Rca structure and function (Zhang et al., 2002).

**TABLE 1:** Rca protein species collected from NCBI database and used to construct the phylogenetic tree.

Accession number	Protein name	Scientific name of each species
XP_005651127.1	Rubisco activase	*Coccomyxa subellipsoidea* C-169
XP_003057993.1	Ribulose biphosphate carboxylase/oxygenase activase, chloroplast precursor	*Micromonas pusilla* CCMP1545
KAF5833834.1	Rubisco activase	*Dunaliella salina*
GBF99182.1	Rubisco activase	*Raphidocelis subcapitata*
AEL29575.1	Chloroplast rubisco activase	*Auxenochlorella pyrenoidosa*
PSC74065.1	Rubisco activase	*Micractinium conductrix*
GAQ85488.1	Ribulose bisphosphate carboxylase/oxygenase activase, chloroplast	*Klebsormidium nitens*
XP_024384352.1	Ribulose bisphosphate carboxylase/oxygenase activase, chloroplastic-like	*Physcomitrium patens*
XP_024396203.1	Ribulose bisphosphate carboxylase/oxygenase activase, chloroplastic-like	*Physcomitrium patens*
XP_024385321.1	Ribulose bisphosphate carboxylase/oxygenase activase 2, chloroplastic-like	*Physcomitrium patens*
XP_024360093.1	Ribulose bisphosphate carboxylase/oxygenase activase 2, chloroplastic-like isoform X2	*Physcomitrium patens*
XP_024360092.1	Ribulose bisphosphate carboxylase/oxygenase activase 2, chloroplastic-like isoform X1	*Physcomitrium patens*
XP_024392724.1	Ribulose bisphosphate carboxylase/oxygenase activase, chloroplastic-like	*Physcomitrium patens*
XP_024544531.1	Ribulose bisphosphate carboxylase/oxygenase activase 2, chloroplastic	*Selaginella moellendorffii*
XP_002982838.1	Ribulose bisphosphate carboxylase/oxygenase activase 2, chloroplastic	*Selaginella moellendorffii*
XP_024521402.1	Ribulose bisphosphate carboxylase/oxygenase activase 1, chloroplastic isoform X4	*Selaginella moellendorffii*
XP_024532198.1	Ribulose bisphosphate carboxylase/oxygenase activase 1, chloroplastic isoform X4	*Selaginella moellendorffii*
XP_024521393.1	Ribulose bisphosphate carboxylase/oxygenase activase 1, chloroplastic isoform X3	*Selaginella moellendorffii*
XP_024532199.1	Ribulose bisphosphate carboxylase/oxygenase activase 1, chloroplastic isoform X5	*Selaginella moellendorffii*
AHL24664.1	Ribulose-1,5-bisphosphate carboxylase/oxygenase activase small isoform	*Pinus massoniana*
AHL24663.1	Ribulose-1,5-bisphosphate carboxylase/oxygenase activase large isoform	*Pinus massoniana*
QKD76840.1	Ribulose bisphosphate carboxylase/oxygenase activase	*Cymbidium tracyanum x Cymbidium iridioides*
XP_004490873.1	Ribulose bisphosphate carboxylase/oxygenase activase, chloroplastic	*Cicer arietinum*
XP_047154954.1	Ribulose bisphosphate carboxylase/oxygenase activase, chloroplastic	*Vigna umbellata*
XP_047181279.1	Ribulose bisphosphate carboxylase/oxygenase activase, chloroplastic-like	*Vigna umbellata*
XP_047174530.1	Low quality protein: Ribulose bisphosphate carboxylase/oxygenase activase 2, chloroplastic-like	*Vigna umbellata*
XP_031743472.1	Ribulose bisphosphate carboxylase/oxygenase activase, chloroplastic isoform X6	*Cucumis sativus*
NP_001267655.1	Ribulose bisphosphate carboxylase/oxygenase activase, chloroplastic	*Cucumis sativus*
XP_011656298.1	Ribulose bisphosphate carboxylase/oxygenase activase, chloroplastic isoform X5	*Cucumis sativus*
XP_031743473.1	Ribulose bisphosphate carboxylase/oxygenase activase, chloroplastic isoform X8	*Cucumis sativus*
XP_011656299.1	Ribulose bisphosphate carboxylase/oxygenase activase, chloroplastic isoform X7	*Cucumis sativus*
XP_004138462.1	Ribulose bisphosphate carboxylase/oxygenase activase, chloroplastic isoform X2	*Cucumis sativus*
XP_031743127.1	Ribulose bisphosphate carboxylase/oxygenase activase, chloroplastic, chloroplastic isoform X1	*Cucumis sativus*
XP_004147680.1	Ribulose bisphosphate carboxylase/oxygenase activase 2, chloroplastic	*Cucumis sativus*
ANH11446.1	Rubisco activase alpha isoform	*Oryza australiensis*
XP_018514080.2	Ribulose bisphosphate carboxylase/oxygenase activase, chloroplastic	*Brassica rapa*
XP_018514080.2	Ribulose bisphosphate carboxylase/oxygenase activase, chloroplastic	*Brassica rapa*
XP_009133378.2	Ribulose bisphosphate carboxylase/oxygenase activase, chloroplastic isoform X1	*Brassica rapa*
XP_033148788.1	Ribulose bisphosphate carboxylase/oxygenase activase, chloroplastic isoform X2	*Brassica rapa*
XP_009143290.2	Ribulose bisphosphate carboxylase/oxygenase activase, chloroplastic isoform X1	*Brassica rapa*
NP_850320.1	Rubisco activase	*Arabidopsis thaliana*
NP_565913.1	Rubisco activase	*Arabidopsis thaliana*

Up until the evolution of polyploidy and flowering plants, the *Rca* gene existed as a single copy that was conserved throughout all species (Roesler and Ogren, 1990; Zarzycki et al., 2013). As plant families and subfamilies diverged, a series of gene and whole genome duplication events occurred (Rensing et al., 2008; Banks et al., 2011), producing multiple *Rca* gene copies in many species (Salvucci et al., 2003; Carmo-Silva et al., 2015). This allowed for more flexibility in the *Rca* gene selection process, which increased variation in Rca structure and function and enabled organisms to adapt to different environments more easily. In some grasses, a tandem gene duplication event occurred before the divergence of the Poaceae family, resulting in tandemly oriented *Rca* genes in later grass species. This discovery helped explain some of the structural and functional differences seen in grass Rca, such as the impact of heat on Rubisco activation (Rundle and Zielinski, 1991). Detailed genetic analysis on Rca can be found in the following review (Nagarajan and Gill, 2018).

Unlike non-green algae (Tabita, 1999), higher plant Rca is a chloroplast enzyme encoded in nuclear genes and synthesized in cytosol (Motohashi et al., 2001), as the small subunits of Rubisco (RbcS) of green algae and plants (Tabita, 1999). However, in eukaryotes, the large subunit of Rubisco (RbcL) is encoded in the chloroplast genome (Tabita, 1999). To form a Rubisco enzyme complex, the translated RbcS is then transported to the chloroplast to associate with RbcL (Dobberstein et al., 1977; Highfield and Ellis, 1978). Higher plant Rubisco assembly and regulation largely depend on the nuclear expression levels for RbcS and Rca.

#### Alternative splicing generates two types of Rca

Among plant Rca, alternative splicing or separation of *Rca* genes generate two types of Rca: long- (α; 46 kDa) and short- (β; 43 kDa) form Rca (Werneke et al., 1989; To et al., 1999). Expression levels of the two isoforms are post-transcriptionally regulated (Perdomo et al., 2021) and can be modulated by environmental changes, such as heat stress (Law and Crafts-Brandner, 2001; Ristic et al., 2009; Wang et al., 2010).

Both isoforms activate Rubisco *in vitro* (Salvucci et al., 2003) and *in vivo* (Zhang et al., 2002). Compared to the short β-isoform, αRca has an additional C-terminal extension (CTE), which contains a redox switch with two specific cysteines (Zhang and Portis, 1999) (Figure 1B). Cyanobacterial Rca contains a rudimentary CTE region, which differs significantly from the redox-sensitive CTE domain in higher plants (Lechno-Yossef et al., 2020). The redox state of the two cysteines is modulated by thioredoxin-mediated dithiol/disulfide exchange (Zhang and Portis, 1999). Because of this, αRca is sensitive to light regulation. This mechanism first appeared during the evolution of alternative splicing mechanisms in charophytes (Nagarajan and Gill, 2018), suggesting it may have been related to new regulatory requirements of land plants. However, unlike Arabidopsis αRca, the reduction of soybean αRca had no impact on Rubisco activity (Harvey et al., 2022). The relationship between light regulation and redox modulation is still unclear.

In some higher plants, species-specific tandem duplication events, intron losses, and random mutations caused changes in alternative splicing mechanisms (Rundle and Zielinski, 1991; Carmo-Silva et al., 2015; Nagarajan and Gill, 2018). As a result, some plants only express one of the two isoforms. For example, several grasses, members of the Solanaceae family, and tobacco plants only express βRca isoform (Zhang and Portis, 1999).

#### Classification of Rubisco and Rca

Rubiscos have a common form established by large catalytic subunit dimers. Based on the sequence similarities between the large subunits, Rubiscos can be categorized into four types: forms I, II, III, and IV (Tabita, 1999; Tabita et al., 2007). Form I is the predominant form with both large (RbcL; ∼50 kDa) and small (RbcS; ∼15 kDa) subunits in plants, eukaryotic algae, cyanobacteria, and some proteobacteria (Tabita, 1999). Form I Rubisco is a hexadecameric cylindrical complex, consisting of eight large subunits and eight small subunits (Taylor and Andersson, 1996). Based on the sequence homology, the group can be further divided into green (IA: Proteobacteria and cyanobacteria; IB: Cyanobacteria and prochlorales) and red- (IC: proteobacteria and chloroflexi; ID: proteobacteria and eukaryotes) types (Delwiche and Palmer, 1996; Watson and Tabita, 1997; Tabita, 1999; Dubbs and Tabita, 2004). Accordingly, Rca can be categorized into three groups: red-type (CbbX), green-type, and CbbQO Rca (Salvucci et al., 1985; Portis, 2003; Mueller-Cajar et al., 2011; Tsai et al., 2015). CbbQO is an activase system that requires an adaptor CbbO and an AAA+ ATPase CbbQ (or CbbQ_6_ hexamer), which are encoded near the sequences coded for Rubisco (Tsai et al., 2015). Unlike red-type Rca, green-type Rca does not function under allosteric regulation by RuBP (Mueller-Cajar, 2017).

Form II, III, and IV Rubiscos do not have small subunits and only consist of large catalytic subunits (Tabita et al., 2008). Form II is established by various numbers of large subunit dimers, which have a distinct catalytic activity compared to Form I (Tabita et al., 2008). The activation of Form II Rubisco has various modes. The activation of Form II Rubisco from *Rhodospirullum rubrum* does not rely on Rca (Jordan and Chollet, 1983; Pearce, 2006). Form II Rubisco of *Acidithiobacillus ferrooxidans* has been found to associate with an activase with a heterooligomer of CbbO and CbbQ_6_ ATPase (Tsai et al., 2015), different from cyanobacterial and plant Rcas. Form III Rubiscos are mostly found in archaea and consist of either a large subunit dimer or a pentamer of large subunit dimers (Tabita, 1999; Watson et al., 1999). Form IV Rubiscos, found in bacteria *Chlorobaculum tepidum* and *Bacillus subtilis*, perform carbon fixation without RuBP (Hanson and Tabita, 2001), and are also known as Rubisco-like proteins (RLPs). Functional analysis linked the RLP function to the enolase reaction (Imker et al., 2007), but the mechanism requires further study. To date, information about the activase for form III or IV Rubisco is limited (Liu et al., 2017).

#### Rubisco activation by Rca is species-specific

Rca regulatory actions are species-specific (Carmo-Silva and Salvucci, 2013). For example, Rca from tobacco and members of the Solanaceae family is ineffective in activating Rubisco from non-Solanaceae family members (Wachter et al., 2013). Hybrid Rubisco composed of the RbcS and RbcL from different species was not activated by tobacco Rca (Wachter et al., 2013). However, Rcas from different species contain common modules for ATP binding and catalysis. The ATP/ADP ratio has been found to have a similar effect on spinach or Arabidopsis Rca (Kallis et al., 2000; Carmo-Silva and Salvucci, 2013), and the Rca AAA+ ATPase modules from different species are conserved in key motifs, such as Walker A and B motifs (Ammelburg et al., 2006). Genetic analysis showed that the ATPase and C-terminal domain of Rca are conserved in sequence among all species, but the N-terminal domains are varied among prokaryotes, cyanobacterium, chlorophyte, and higher plants (Nagarajan and Gill, 2018). Currently available Rca structures also showed a conserved structural motif of the ATPase module. (Mueller-Cajar et al., 2011; Stotz et al., 2011; Sutter et al., 2015; Flecken et al., 2020; Tsai et al., 2020). Sequence comparison suggested that the N-terminal domain was gained when the Rca coding sequence migrated to the nuclear genome, where sequence divergence or domain rearrangements potentially occurs (Nagarajan and Gill, 2018). This may result in functional diversification on species-specific activation on Rubisco by Rca for adapting to environmental changes (Hanson and Whiteheart, 2005; Sysoeva, 2017). Further understanding will require functional characterizations and systematic studies on the N-terminal Rca.

### Structure and function of Rca

Rca is a member of the Type I AAA+ (ATPases associated with diverse cellular activities) protein superfamily, and it contains one ATPase module (Bhat et al., 2017b) (Figure 1B). AAA+ ATPase superfamily members are involved in various biological functions that require ATP hydrolysis, such as segregation of ubiquitylated proteins from their original cellular compartments, unfolding proteins for degradation, and remodeling proteins for enzymatic activation (Snider et al., 2008; Olivares et al., 2016). AAA+ ATPases usually form a hexameric ring, allowing the interacting substrate to bind in the ring center.

Rca consists of an N-terminal chloroplast transit peptide (cTP), an N-terminal regulatory domain, an ATPase module, a Rubisco recognition domain, and a C-terminal domain (Portis et al., 2008) (Figure 1B). αRca contains an additional CTE domain at C-terminus (Zhang and Portis, 1999) (Figure 1B). The cTP and N-terminal domain are missing from prokaryotic species, such as *Halothiobacillus neapolitanus*, but not from higher chlorophytes or plants (Nagarajan and Gill, 2018) (Figure 1B). The first appearance of these structures correlated with the *Rca* gene’s migration to the nucleus during chlorophyte evolution (Archibald, 2009; Keeling, 2010), suggesting they play a necessary role in transporting Rca to the chloroplast where Rubisco activation takes place. However, the role of the N-terminal domain in Rca transport is still unclear. The N-terminal domain was suggested to be structurally flexible (Blayney et al., 2011; Stotz et al., 2011; Keown and Pearce, 2014). Because of its high structural mobility, it is challenging to obtain full-length structural information using current structural methods. The N-terminal domain interacts directly with Rubisco, and residues within it play a role in Rubisco activation (van de Loo and Salvucci, 1996). Thus, it is unclear how Rca activates Rubisco in prokaryotes, where no regulatory N-terminal domain is present.

The ATPase module of Rca is conserved with those across species in the AAA+ superfamily. The ATPase module has an N-terminal α/β-nucleotide-binding subdomain and a C-terminal α helical subdomain (Shivhare and Mueller-Cajar, 2017) (Figure 1C). Nucleotide-binding sites are at the interface between monomers. It is hypothesized that the ATPase modules hydrolyze ATP and utilize energy to change the conformation and generate a force to pull the substrate. This substrate threading model has been proposed in other AAA+ ATPases (Rizo et al., 2019; Twomey et al., 2019; Ripstein et al., 2020).

Redox modulation on the CTE regulates Rca activity. Although the structural evidence for the mechanism of redox modulation is still lacking, the site-directed mutagenesis and cross-linking experiment suggested a model in which the negatively charged residues of the CTE alter the ADP sensitivity of the ATPase module (Zhang and Portis, 1999; Wang and Portis, 2006; Portis et al., 2008). The model suggested that the reduced form of αRca favors ATP binding (Zhang et al., 2001; Wang and Portis, 2006). The cross-linking experiment indicated that in the oxidized form, these negatively charged residues are close to the nucleotide-binding site and can interact with surrounding positively charged residues through disulfide bond formation, thereby interfering with ATP binding (Zhang et al., 2001; Wang and Portis, 2006). Unfortunately, the structural model is still lacking in the mechanistic information about this redox modulation.

#### Rca assembly state is highly polydisperse and dynamic, with hexamer being an important functional form

Recent structural models show that Rca is functional in a hexameric form (Figure 1D) (Mueller-Cajar et al., 2011; Stotz et al., 2011; Hasse et al., 2015; Flecken et al., 2020). Mutagenesis studies and the crystal structure of the N- and C-terminally truncated Rca showed that the hexameric formation is mainly mediated through its ATPase module (Stotz et al., 2011). On the other hand, Rca assembly state is highly polydisperse in solution. Species ranging from monomers to hexamers, as well as larger aggregates, were frequently resolved in traditional biophysical and biochemical assays in a concentration dependent manner (Henderson et al., 2013; Keown et al., 2013). Early investigations using intrinsic fluorescence and gel filtration chromatography suggested that the oligomerization of Rca is nucleotide-dependent (Wang et al., 1993). Analytical ultracentrifugation result showed that the ATP promotes higher oligomeric formation, such as tetramer and hexamer, from dimers, for spinach Rca (Keown and Pearce, 2014).

To avoid fractionation and enable characterization under equilibrium conditions, fluorescence correlation spectroscopy (FCS), together with detailed modeling, has been successfully used to investigate Rca assembly (Chakraborty et al., 2012). FCS experiments on tobacco βRca is consistent with a monomer-dimer-tetramer-hexamer assembly pathway (Chakraborty et al., 2012). Follow up experiments on cotton βRca confirmed that ATPγS promotes hexamerization and suggested that free magnesium ion (Mg^2+^) also facilitates hexamer formation but at the expense of the Rca forming larger complexes. (Kuriata et al., 2014). It is suggested that Mg^2+^-mediated regulation may be related to the light-dark adaptation of the photosynthetic system *in vivo* (Hazra et al., 2015). RuBP is also found to be a critical allosteric regulator for some Rca, such as Rca in *Rhodobacter sphaeroides*, and can stabilize them in a hexameric form (Mueller-Cajar et al., 2011). Most recently, comprehensive FCS experiments, together with ATPase activity assays on tobacco Rca, revealed that peak catalytic rate (at between 0.5 and 2.5 μM Rca), coincide with an Rca composition containing significant amount of coexisting dimers, tetramers, and hexamers (Serban et al., 2018).

**FIGURE 2:**
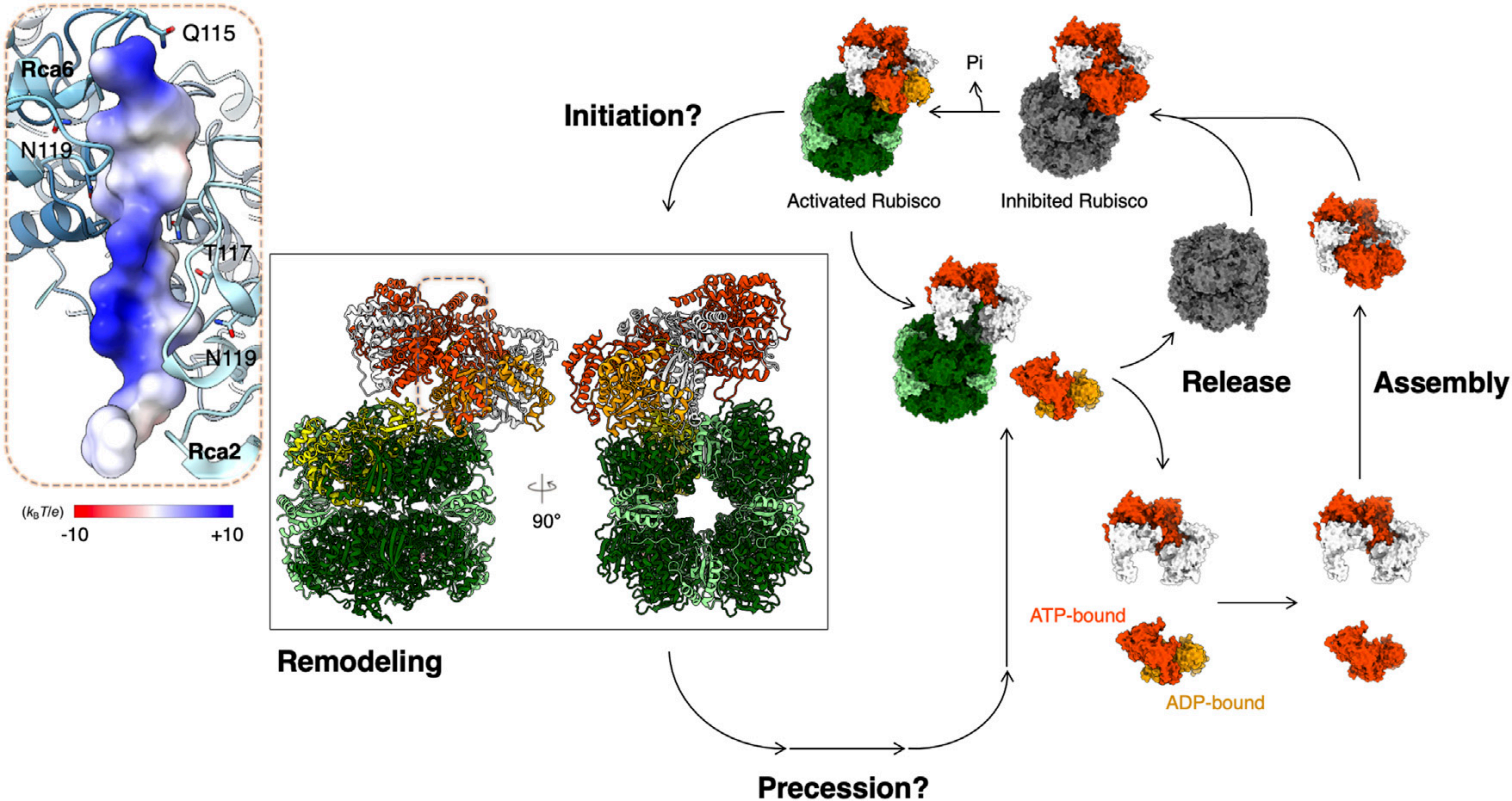
Proposed model for Rubisco activation by Rca. Structural models are presented for Rubisco and Rca from *Nostoc* sp. PCC 7120 (PDB code: 6Z1F) (Flecken et al., 2020). Color codes for atomic model: RbcS—light green; RbcL—dark green; N-terminal pulled RbcL—yellow; CAP—pink; ADP—orange; ATPγS—orange-red. Rca is in an equilibrium of multiple oligomeric forms before binding onto Rubisco. The Rca assembly life cycle depends on the nucleotide-binding states (Serban et al., 2018). ATP- and ADP-bound Rca in its assembly life cycle are presented in orange red and orange, respectively. Inset: Bound N-terminus of RbcL is shown in surface representation colored according to its Coulomb potential.

Recently, single-molecule diffusometry experiments shed new light on the oligomerization behavior of tobacco βRca (Wang et al., 2018). This assay uses an Anti-Brownian ELectrokinetic (ABEL) trap to measure the diffusion coefficient of individual protein complexes in solution and builds up full distributions of assembly states under equilibrium conditions (Wang and Moerner, 2014). These experiments confirmed that assembly of *Nt*βRca is nucleotide dependent, directly resolved the monomer-dimer-tetramer-hexamer assembly pathway and revealed that large oligomers (here tetramers and hexamers) assemble cooperatively in the presence of ATPγS as the nucleotide. Further, by analyzing the single-molecule traces, assembly-disassembly events can be monitored in real-time. It was found that the major difference between ATPγS-bound and ADP-bound Rca is the *dynamics* of subunit exchange: ADP-bound Rcas were observed to assemble and disassemble rapidly (∼0.3 s^-1^ at 8 µM Rca), while with ATPγS, subunit exchange was rare and at least 2-3 fold slower (Wang et al., 2018). These observations, together with parallel FCS experiments, suggest an alternative model of Rca function, in which ATP binding and hydrolysis are coupled to subunit assembly/disassembly (Serban et al., 2018) (Figure 2). Function of Rca is not associated with one particular assembly state but involves dynamic cycling through dimer, tetramer and hexamer forms (Figure 2). Further testing of this model using single-molecule and traditional approaches are underway in the author’s laboratories.

The dynamic engagement of Rca with Rubisco and how it is affected by Rca oligomerization is not fully understood, the hexameric organization has been shown to be important in the arrangement of the Rca central loops required for Rubisco remodeling (Stotz et al., 2011; Shivhare et al., 2019; Flecken et al., 2020; Ng et al., 2020). It is hypothesized that hexamers play a critical role in substrate pulling force, which requires energy from ATP hydrolysis (Mueller-Cajar et al., 2011; Stotz et al., 2011; Loganathan et al., 2016).

#### Rca is temperature-sensitive

Rca has been shown to be thermolabile (Robinson et al., 1988; Crafts-Brandner and Salvucci, 2000; Salvucci and Crafts-Brandner, 2004). High temperature was found to be the primary contributing factor in impairing Rubisco activation by Rca (Crafts-Brandner and Salvucci, 2000; Salvucci and Crafts-Brandner, 2004; Kim and Portis, 2005; Galmés et al., 2013). The expression levels of the α and β isoforms are also varied under heat stress in rice (Wang et al., 2010), wheat (Law and Crafts-Brandner, 2001), and maize (Ristic et al., 2009). Higher expression levels of the α isoform may help mediate stress from high temperatures and regulate the Rubisco activity in some plant cells. A recent study on rice has shown that the increasing levels of Rubisco and Rca improve photosynthesis only within a specific range of temperatures (Qu et al., 2021; Suganami et al., 2021). It has been found that a single amino-acid mutation (M159I) on the Rca ATPase domain can vary the thermosensitivity of Rca in *Triticum aestivum* (Degen et al., 2020). Also, triple mutants of Arabidopsis Rca (F168L |V257I |K310N and M131V |V257I |K310N) showed a higher thermostability with a 10°C increase (Kurek et al., 2007). These results open an opportunity to engineer Rca as a heat-resistant enzyme for activating Rubisco under high temperatures (Qu et al., 2023).

### Mechanism of Rubisco activation by Rca

Rca catalyzes the carboxylation of RuBP and generates two 3PGA molecules in the CBB cycle (Bassham et al., 1950). To be activated, a specific lysine at Rubisco’s active site needs to be carbamylated with a bound Mg^2+^ ion (Stec, 2012). The current model of Rubisco activation by Rca involves changing the conformation of this active site (Bhat et al., 2017a). Two hypothetical models for the binding of Rca for Rubisco activation have been suggested. One binding mode involves stacking of the Rca hexameric ring in a way such that the four-fold ring of the Rubisco holoenzyme and the six-fold axis of Rca are aligned. This model involves molecular contacts between Rca and the RbcS through a ring-ring stacking mechanism, the so-called “top-on” binding mode (Wachter et al., 2013). The other binding mode is the “side-on” model, in which the Rca hexameric toroid binds Rubisco by aligning its central pore over the two-fold axis of a functional large-subunit dimer (Wachter et al., 2013). The side-on binding positions two active sites and two recognition elements of Rubisco near the central pore edge of the Rca hexamer. In this side-on spatial arrangement, Rca makes contact with the RbcL. In addition to Rca binding, magnesium has also been reported to be involved in Rubisco activation under high-temperature stress (Shao et al., 2021).

#### Structure of Rubisco-Rca complex

The hexameric form of Rca is critical in activating Rubisco, and it is mainly driven by the Rca AAA+ ATPase domains (Stotz et al., 2011; Tsai et al., 2020). Mutagenesis studies have shown that the N-terminal domain of the Rca is important to Rubisco activation and does not regulate ATPase activity (van de Loo and Salvucci, 1996). It is still unknown how the N-terminus of Rca participates in Rubisco activation or how Rca initializes the activation. Cryogenic electron microscopy (cryo-EM) has recently revolutionized the study of high-resolution structures of biological macromolecules and protein complexes (Kühlbrandt, 2014). Multiple AAA+ ATPase cryo-EM structures have indicated a possible model for substrate processing (Gates and Martin, 2020), and because Rca AAA+ ATPase is a member of the AAA+ superfamily, the working mode of the Rca on activating Rubisco is likely to be conserved across other superfamily members.

Currently available cryo-EM structures of the Rubisco-Rca complexes show possible functional modes in different species. A low-resolution cryo-EM structure of the red-type Rca from *R. sphaeroides* shows Rca binding on one Rubisco active site, possibly engaging the C-terminal strand of the RbcL (Bhat et al., 2017a). However, the form IB Rubisco lacks the extended C-terminal sequence of the RbcL (Tabita et al., 2008), implying it must have a different mode of engagement between Rubisco and Rca.

In some species, the N-terminal RbcL seems to interact with Rca. A mutagenesis study of the RbcL of *Arabidopsis* Rubisco showed that Rca function is sensitive to the N-terminal RbcL for activating Rubisco (Ng et al., 2020). A cryo-EM structure of the cyanobacterial Rubisco-Rca complex of *Nostoc* sp. was determined and showed that the Rca binds on the side of the Rubisco complex and pulls and denatures the N-terminus of RbcL through its central hole (Figure 2) (Flecken et al., 2020). Although the mechanism of how the Rca recognizes the N-terminal RbcL is still unclear, from what is known of the general mechanism of the AAA+ ATPase enzymes, the Sensor 2 motif may play an important role in recognition (McAlear et al., 1994; Smith et al., 1999). Although this structural evidence begins to reveal the Rubisco reactivation by Rca, the mechanistic details of the initiation, precession, and recovery remain unclear (Figure 2). More structural evidence is required to answer these questions and provide mechanistic insight into Rubisco activation by Rca.

## Concluding remarks

Due to the dramatic climate changes, we have an urgent need to either increase crop production or improve the efficiency of carbon fixation to accommodate lowering atmospheric CO_2_. Engineering Rubisco’s active site to improve its carboxylation efficiency is one direction to improve photosynthesis (Iñiguez et al., 2021). The temperature-sensitive property of the Rca can also be considered in engineering a crop to adapt to climate change. Overexpression of Rubisco in rice showed no significant improvement in photosynthetic efficiency (Makino and Sage, 2007; Suzuki et al., 2009). However, Rca overexpression enhanced photosynthetic efficiency in bacteria (Gunn et al., 2020) and showed a higher crop yield in rice (Wu et al., 2007; Fukayama et al., 2012), wheat (Ristic et al., 2009), soybean (Yin et al., 2010; Chao et al., 2014), and maize (Morales et al., 1999; Wang et al., 2021). Recent development on including Rca into carboxysomes shows a promising route to improve carbon-fixing efficiency (Chen et al., 2022; Tsai et al., 2022). Thus, enhancing our understanding of the molecular mechanism of Rubisco reactivation by Rca is critical in helping us improve the crop production, either by engineering a highly efficient enzyme or modulating the enzyme function. More details for using Rca as a target to improve crop production can be seen in this review (Wijewardene et al., 2021). It is imperative to acquire this piece of knowledge, which can be applied in crop improvements and carbon fixation efficiency in response to climate changes.

## Abbreviations

AAA+, ATPases associated with diverse cellular activities; CBB cycle, Calvin-Benson-Bassham cycle; CTE, C-terminal extension; Rca, rubisco activase; Rubisco, ribulose-1,5-bisphosphate carboxylase-oxygenase.
